# Development and Verification of a Novel Three-Dimensional Aqueous Outflow Model for High-Throughput Drug Screening

**DOI:** 10.3390/bioengineering11020142

**Published:** 2024-01-31

**Authors:** Matthew Fung, James J. Armstrong, Richard Zhang, Anastasiya Vinokurtseva, Hong Liu, Cindy Hutnik

**Affiliations:** 1 Schulich School of Medicine & Dentistry, Western University, London, ON N6A 3K7, Canada; 2 Department of Ophthalmology, Schulich School of Medicine & Dentistry, Western University, London, ON N6A 3K7, Canada; 3 Department of Ophthalmology, Ivey Eye Institute, St. Joseph’s Health Center, London, ON N6A 4V2, Canada

**Keywords:** glaucoma, intraocular pressure, microfluidics, inflammation, fibrosis, tissue-mimetic, drug screening, organ modelling, three-dimensional aqueous outflow

## Abstract

Distal outflow bleb-forming procedures in ophthalmic surgery expose subconjunctival tissue to inflammatory cytokines present in the aqueous humor, resulting in impaired outflow and, consequently, increased intraocular pressure. Clinically, this manifests as an increased risk of surgical failure often necessitating revision. This study (1) introduces a novel high-throughput screening platform for testing potential anti-fibrotic compounds and (2) assesses the clinical viability of modulating the transforming growth factor beta-SMAD2/3 pathway as a key contributor to post-operative outflow reduction, using the signal transduction inhibitor verteporfin. Human Tenon’s capsule fibroblasts (HTCFs) were cultured within a 3D collagen matrix in a microfluidic system modelling aqueous humor drainage. The perfusate was augmented with transforming growth factor beta 1 (TGFβ1), and afferent pressure to the tissue-mimetic was continuously monitored to detect treatment-related pressure elevations. Co-treatment with verteporfin was employed to evaluate its capacity to counteract TGFβ1 induced pressure changes. Immunofluorescent studies were conducted on the tissue-mimetic to corroborate the pressure data with cellular changes. Introduction of TGFβ1 induced treatment-related afferent pressure increase in the tissue-mimetic. HTCFs treated with TGFβ1 displayed visibly enlarged cytoskeletons and stress fiber formation, consistent with myofibroblast transformation. Importantly, verteporfin effectively mitigated these changes, reducing both afferent pressure increases and cytoskeletal alterations. In summary, this study models the pathological filtration bleb response to TGFβ1, while demonstrating verteporfin’s effectiveness in ameliorating both functional and cellular changes caused by TGFβ1. These demonstrate modulation of the aforementioned pathway as a potential avenue for addressing post-operative changes and reductions in filtration bleb outflow capacity. Furthermore, the establishment of a high-throughput screening platform offers a valuable pre-animal testing tool for investigating potential compounds to facilitate surgical wound healing.

## 1. Introduction

The efficacy of glaucoma surgery hinges on sustained improvements in aqueous humor (AH) outflow to control intraocular pressure (IOP), and, consequently, further glaucoma progression. AH outflow is a critical factor influenced by iatrogenic tissue damage during surgery. Following the surgical insult, damaged tissue initiates a wound healing response, exposing the subconjunctiva to acute surgical inflammation and endogenous pro-inflammatory and pro-fibrotic cytokines in the AH [1]. This pro-inflammatory cascade contributes to excessive wound healing and post-operative subconjunctival fibrosis, ultimately impairing outflow capacity.

A key player in this fibrotic cascade is the transforming growth factor-beta (TGFβ) family of AH cytokines. Present at elevated levels in AH of glaucoma patients [2,3], TGFβ induces alterations in the extracellular matrix (ECM) of the trabecular meshwork, resulting in heightened resistance to aqueous outflow and subsequently elevated intraocular pressure (IOP) [4,5,6]. Beyond the deleterious microenvironmental effects of TGFβ, similar pathophysiological changes can be observed post-operatively in the filtration bleb [7,8]. Thus, specific targeting of cellular processes downstream of TGFβ may be an avenue to mitigate fibrotic processes contributing to surgical failure while preserving processes essential for normal wound healing and homeostasis.

Myofibroblasts, characterized by the increased expression of α-smooth muscle actin (α-SMA), which is stimulated via the SMAD 2/3 pathway downstream of TGFβ, play a crucial role in wound healing [9,10]. This fibroblast-to-myofibroblast transdifferentiation leads to the formation of actin–myosin stress fiber bundles akin to those found in muscle cells, earning them the moniker myofibroblasts. These cells exhibit increased ECM secretion and contractile activity, critical in normal wound-healing processes [11]. After healthy wound healing, myofibroblasts are deactivated or removed via apoptosis. However, in bleb-forming procedures, excessive myofibroblast activity can be harmful, contributing to scar tissue formation and impairing outflow tract patency, both of which contribute to surgical failure [12,13,14]. Thus, drugs targeting TGFβ-mediated myofibroblast activity may help maintain outflow patency throughout the post-operative period.

Despite substantial advancements in surgical glaucoma intervention, progress in developing compounds to address post-operative fibrosis has been slow [15,16]. Current strategies rely on local anti-metabolite treatments, such as mitomycin C (MMC) or 5-fluorouracil (5FU). However, their short half-lives may yield insufficient anti-fibrotic efficacy and the non-specific targeting of all cell types often leads to undesirable off-target effects, including tissue thinning, leaks, and endophthalmitis [17,18]. Additionally, ocular steroid therapy, used to control post-surgical inflammation, poses a major risk factor for cataracts and paradoxically increases the risk of steroid-induced glaucoma [19]. Overall, these interventions carry significant risks, necessitating the exploration of novel avenues to address inflammatory changes after surgery.

The current drug development process begins with 2D cell culture and progresses to animal models. However, cells in 2D cell culture are separated from physiological factors affecting cellular behavior. In the eye, fibroblasts are typically associated with an ECM that modulates cell growth and activity [20]. Additionally, fibroblasts are sensitive to the “stiffness” of the surface on which they are grown, where solid plastic culture plates have been known to inherently induce myofibroblast transdifferentiation, potentially confounding experimental results [21,22,23]. Conversely, animal models do account for the physiological context of fibroblasts within a perfused organ system. However, these experiments tend to have significant ethical and financial cost associated with them, are significantly more time-consuming than in vitro work, and, as such, have challenges that prevent them from being applicable in the early drug screening process. In addition, interspecies variability cannot fully mimic the conditions observed in humans [24,25].

Thus, the purpose of this study is to elucidate the effects of TGFβ on AH outflow capacity in distal outflow bleb-forming procedures. We aimed to adapt previous methods of in vitro blood vessel modeling [26] to develop a high-throughput drug testing platform that better mirrors the subconjunctival environment to screen potential post-surgical anti-fibrotic adjuvants. The current study introduces a novel perfused 3D collagen-based subconjunctival tissue mimetic to assess the effects of TGFβ and fibrosis-modulating treatments on AH outflow. In doing so, the model aims to better replicate the physiological milieu afforded by animal models while providing a cost-effective and high-throughput in vitro testing platform. This study hypothesizes that exogenous TGFβ1 will increase outflow resistance within this novel in vitro model and using a small molecule to inhibit a secondary messenger downstream of the TGFβ1 pathway will mitigate this effect. 

## 2. Methods

### 2.1. Primary Human Tenon’s Capsule Fibroblast (HTCF) Procurement and Culture

**Figure 1:**
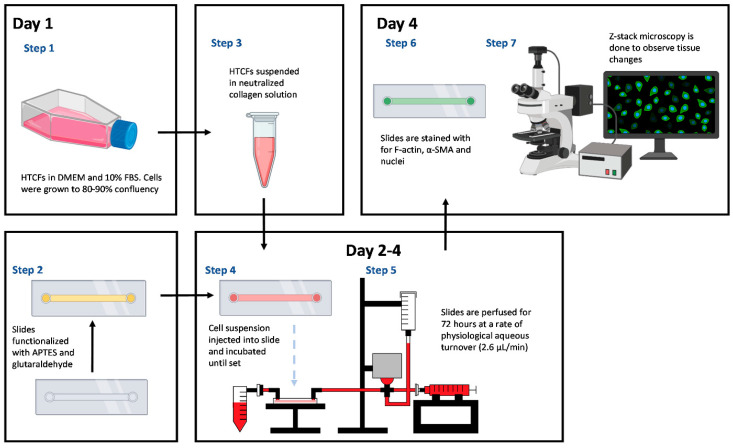
**Microfluidic workflow:** Pre-clinical applicability is assessed through pressure changes throughout the experiment within the microfluidic model. Fluorescent microscopy was used to link morphological changes in hydrogel to functional changes in the model.

Primary HTCFs were obtained from 2–4 mm^3^ resections of Tenon’s capsule during ocular surgeries at the Ivey Eye Institute, London, ON, Canada, before anti-metabolite treatment [27]. This study adhered to the tenets of the Declaration of Helsinki. Ethical approval (HSREB: 106783) was obtained for patient demographic data collection associated with tissue explants. Informed consent was secured before tissue procurement, with no alteration to patient surgeries. Samples were cultured in Dulbecco Modified Eagle’s Minimum Essential Medium (DMEM, Gibco, Waltham, MA, USA, Cat# 12430054), supplemented with 10% Fetal Bovine Serum (FBS, Gibco, Waltham, MA, USA, Cat# A38403), 2mM L-glutamine (Gibco, Waltham, MA, USA, Cat# A2916801), 1% amphotericin (Gibco, Waltham, MA, USA, Cat# 15290018), and 1% penicillin/streptomycin (P/S, Gibco, Waltham, MA, USA, Cat# 15240096) from Sigma-Aldrich (Oakville, ON, Canada). Fibronectin (Sigma-Aldrich) was used to coat 6-well culture plates (9.5 cm^2^) for seven days. Migrating fibroblasts were trypsinized and stored in liquid nitrogen. HTCFs were incubated in DMEM with 10% FBS at 37 °C in a 5% CO_2_ atmosphere until experimental use or up to the 5th cell passage (Figure 1).

### 2.2. Microfluidic Slide Chamber Preparation

**Figure 2:**
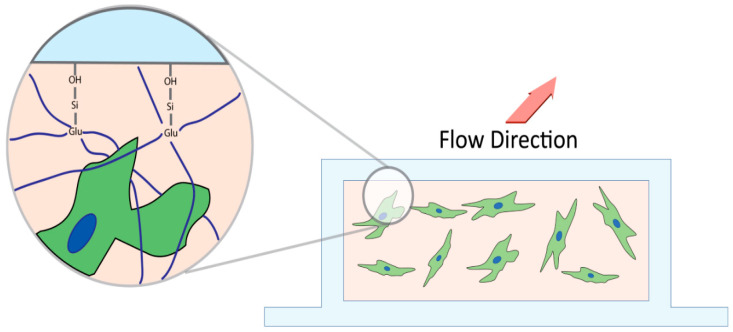
**Cross section schematic of microfluidics chamber.** APTES salinizes the hydroxide groups on the slide, which then allows glutaraldehyde to bind with the silicon group. Glutaraldehyde crosslinks with the loose unincorporated collagen (navy) anchoring it into the microfluidics chamber to prevent extrusion of matrix and Tenon’s capsule fibroblast upon perfusion.

To covalently link collagen to the slide chamber, a µ-slide I microfluidic slide (Ibidi #80176 Fitchburg, MA, USA) was salinized with vaporized 3-Aminopropyl-triethoxysilane (APTES) (Sigma-Aldrich, Oakville, ON, Canada). APTES was heated to 150 °C, and the fumes were pushed through the slide with a syringe pump (Harvard apparatus) for one hour at a rate of 60 mL/h. Unfunctionalized APTES was purged with 10 mL PBS (pH 7.4). After salinization, glutaraldehyde was bonded to the APTES by incubating a 6% glutaraldehyde (Sigma-Aldrich, Oakville, ON, Canada) solution for 30 min at ambient temperature, followed by washing with 10mL PBS (Figure 2). Finally, the chamber was sterilized by perfusing 70% ethanol followed by PBS before manipulation in a sterile hood [28,29].

### 2.3. HTCF Preparation for Hydrogel Formation

HTCFs were grown until 90–100% confluent in a 25 mL Falcon flask, dissociated using trypsin (Fisher Scientific, Waltham, UK), and quantified. The pellet was resuspended in DMEM supplemented with 2% FBS to a concentration of 6.25 × 10^4^ cells/µL. To create the hydrogel, 80 µL of rat tail collagen solution (1.8 mg/mL), 8 µL Waymouth media (Gibco, Cat# 15290018), and 8 µL sterile NaOH solution (0.275M) were mixed, and 4 µL of the cell suspension was added [27]. A total of 50 µL of the collagen–HTCF liquid hydrogel was cast into the functionalized microfluidics slide and incubated at 37 °C in 5% CO_2_ for 30 min until solidified. The final HTCF concentration within the hydrogel was 2.5 × 10^6^ cells/mL.

### 2.4. Perfusion Track Construction and Experimental Setup

**Figure 3:**
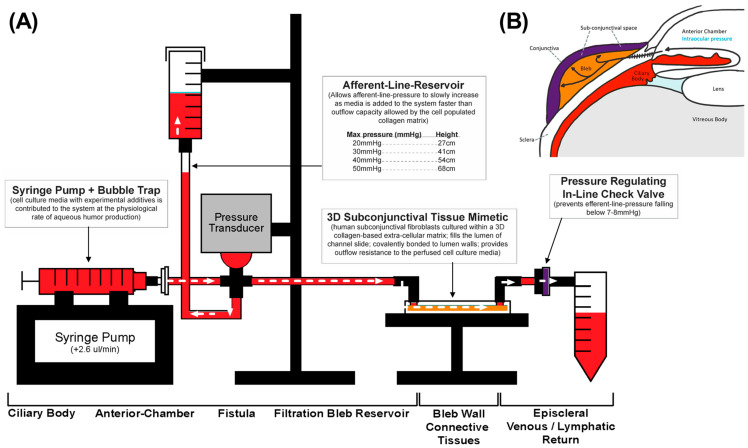
**Microfluidics model** (**A**) **mimics anatomical qualities of surgical filtration bleb** (**B**)**.** Perfusate from the syringe pump mimics aqueous humor production in the ciliary bodies (red). The surgical filtration bleb (orange) is modelled by the collagen hydrogel in the slide chamber. Finally, a pressure gated one-way valve is down located downstream of the slide chamber to mimic episcleral back pressure (purple). As the cells within the hydrogel proliferate, hydrodynamic changes lead to impaired flow. Consequently, perfusate accumulates in the influent pressure column, which can be read manually or measured through the pressure transducer upstream representing IOP.

The model utilized Sanipure BDP 1/8′ ID × 1/4′ OD tubing (Cole-pharma, Quebec, QC, Canada) and polypropylene connectors (Harvard Apparatus, Saint-Laurent, QC, Canada) to construct the track (Figure 3). The track connected the syringe pump to the blood pressure transducer and the tissue slide. Connectors and tubing were autoclaved before assembly. The perfusate in the syringe pump comprised DMEM with 2% FBS, 1% P/S, and 0.25 mmol/L ascorbic acid 2-phosphate (AA2P). Experimental compounds were added to the perfusate according to the treatment groups. The track was primed with media, and slides were connected bead-to-bead on the influent and effluent side to prevent air bubbles.

Two runs were set up in parallel to account for pump error, allowing for paired comparison. For each run, equal lengths of tubing were measured to account for interference from tubing friction. To test the tissue mimetic’s ability to modulate outflow resistance, exogenous TGFβ1 at a concentration of 2 ng/mL was added to the perfusate to simulate post-surgical aqueous humor conditions. This was compared to the tissue-mimetic perfused with vehicle perfusate. To inhibit TGFβ1 mediated SMAD2/3 activity, TGFβ1 perfusate was co-supplemented with 10 µM verteporfin (Sigma Aldrich, Oakville, ON, Canada), and this was compared to the TGFβ1 only group.

### 2.5. Afferent Line Pressure Analysis

Afferent line pressure, serving as an analogue for intraocular pressure (IOP), was measured in real-time with a blood pressure transducer (Harvard Apparatus 72-4498). The pressure was measured at a rate of 10 readings per second, recorded on Labchart 8 (AD instruments). Raw data were down-sampled by 600 times to provide average pressure readings every minute. Baseline afferent pressure was established by averaging the readings in the first hour to account for system pressure equilibration (P_equil_). Every subsequent pressure value was normalized to P_equil_ to give pressure changes relative to baseline (ΔP). ΔP for vehicle control (ΔP_v_) and TGFβ1 (ΔP_t_) were compared to test whether TGFβ1mediated changes to outflow resistance occurred. Differences in ΔP for TGFβ1+ verteporfin treatment (ΔP_vert_) and ΔP_t_ were used to examine verteporfin’s ability to attenuate TGFβ1mediated pressure changes. Afferent line pressure changes served as the primary outcome to test whether a compound may have potential clinical applicability.

### 2.6. Tissue-Mimetic Fixation and Histological Staining

At the end of perfusion experiments, the tissue-mimetic was washed with PBS at 2.6 µL/min. The matrix was fixed by perfusing a 4% paraformaldehyde (Sigma, Oakville, ON, Canada) solution [26] and blocked with a 2% bovine serum albumin (Sigma, Oakville, ON, Canada) solution by perfusing tissue-mimetics for 1 h at 2.6 µL/min. Subsequently, 50 µL of anti-actin, α-smooth muscle—Cy3 mouse monoclonal antibody (5 µg/mL, C6198, Sigma-Aldrich) was pushed into the slide chamber and allowed to incubate at 4 °C overnight. Finally, the slide was stained with 50 µL of F-actin cytopainter (Abcam, Waltham, UK) and 50 µL Hoechst (Sigma-Aldrich, Oakville, ON, Canada) for 30 min and 10 min, respectively, at ambient temperature.

### 2.7. Confocal Immuno-Fluorescent Microscopy

Z-stack microscopy was performed from the upper to the lower tissue margin of the slide to assess three-dimensional structure. Z-stack steps were separated at intervals of 2.54 µm for a total height of 200 µm. Analysis of Z-stack was performed on ImageJ. The number of cells was quantified by projecting the Hoechst (nuclear) channel into various 2D projections and using the particle measurement tool to identify shapes measuring at least 10 × 10 pixels to quantify the number of nuclei visible. For FITC (F-actin) channels, each slide within the stack was thresholded, and the area of staining was measured. The average cellular content of F-actin was calculated by summing the total area of staining for each slide in the FITC channels and dividing by the number of nuclei detected from the DAPI channel. This allowed the comparison of average F-actin expression on a per-nucleus basis to estimate the degree of myofibroblast transdifferentiation.

### 2.8. Western Blot

HTCFs were cultivated to confluency in 6-well plates, subjected to a 24-h serum starvation period, and treated with varied concentrations of verteporfin (Sigma Aldrich) up to 20 µM. Cells underwent a 48-h treatment period and were subsequently lysed in 250 μL of lysis buffer (PhosphoSafe Extraction Reagent, Novagen, Oakville, ON, Canada) supplemented with a protease inhibitor cocktail (P2714, Sigma-Aldrich, Oakville, ON, Canada).

The raw lysate underwent homogenization through a 27 G needle, followed by centrifugation at 13,000× *g* for 10 min to extract the supernatant. Protein content normalization was achieved using a Pierce BCA protein assay kit (ThermoFisher, Rockford, IL, USA), to enable loading of 10 μg of protein per well in a 10% polyacrylamide gel. Electrophoresis ensued for 1 h, and an iBlot Gel Transfer Device (Invitrogen, Rockford, IL, USA) facilitated protein transfer onto a nitrocellulose membrane.

The membrane was subsequently blocked with 5% (*w*/*v*) bovine serum albumin (Sigma-Aldrich, Oakville, ON, Canada) solubilized in Tris-buffered saline (TBST) at ambient temperature for 1 h. Afterwards, the membranes were incubated overnight at 4 °C involving primary antibodies (0.5 µg/mL) against αSMA (ab5694, Abcam, Waltham, UK) and GAPDH (Santa Cruz Biotechnology, Inc., Dallas, TX, USA), diluted in TBST containing 5% BSA (w/vol). Washed membranes were then incubated with 1:3000 (*v*/*v*) dilutions of goat anti-rabbit.t IgG conjugated with horseradish peroxidase (Santa Cruz Biotechnology, Inc., Dallas, UK).

Protein loading control utilized WesternBright Quantum chemiluminescent reagent (Advansta, Inc., San Jose, UK) molecular markers in conjunction with GAPDH. Densitometric analysis and imaging were conducted using the ChemiDoc MP System (Bio-Rad Laboratories, Inc., Hercules, UK) connected to Image Lab (Version 6, Bio-Rad).

## 3. Results

### 3.1. Analysis of Three-Dimensional Tissue Mimetic Structure

**Figure 4:**
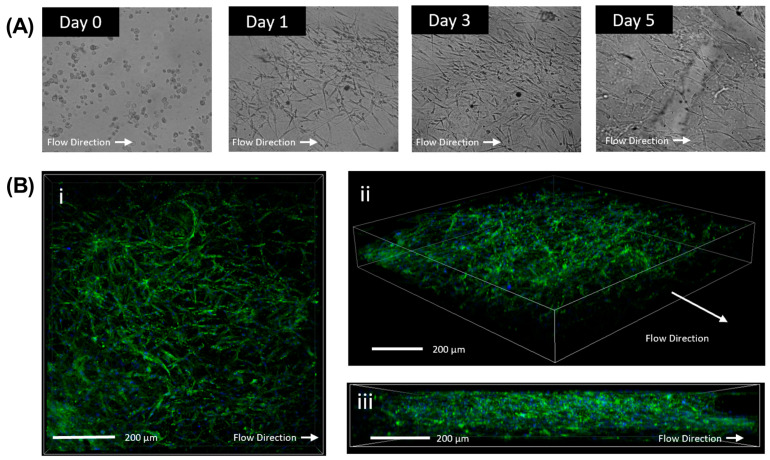
**Verification of three dimensional proliferation of cells within the hydrogel**. Light microscopy of HTCFs within the collagen matrix perfused with perfusion media. Cells demonstrated growth and proliferation within the slides (**A**). Confocal microscopy of slide after being perfused in the model for 72 h. Cells were stained for F-actin stress fibres (FITC) and Hoest (Nuclear). HTCFs demonstrate proliferation within the length (**B**) (i) width (ii), and height (iii) of the flow chamber demonstrating growth three dimensionally.

To assess cellular morphology and integration into the collagen matrix, slides were imaged with light microscopy daily at pre-set regions of interest to observe cellular morphology within the hydrogel. HTCFs within the chamber were observed for at least 5 days within the chamber with maximum cell density achieved approximately at days 3 to 4 (Figure 4A). Z-stack images confirmed hydrogel integrity after the 5-day run and the presence of three-dimensional proliferation of HTCFs throughout the collagen matrix (Figure 4B).

### 3.2. Morphological Changes Due to TGFβ1 and Verteporfin Co-Treatment

**Figure 5:**
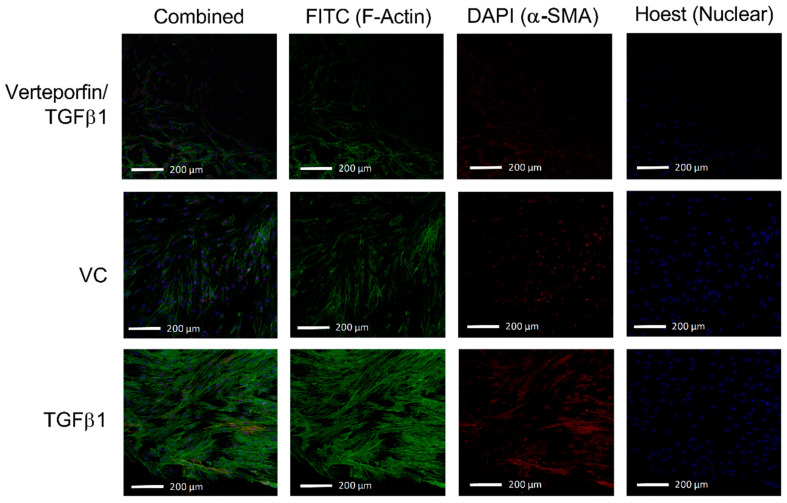
**TGFβ1 leads to increased expression of F-actin stress fibres and α-SMA.** Represented is an overlay of all 2.54 µm images within the Z-stack overlayed into a two-dimensional image to depict differences in fluorescent intensity between groups following the 72-h treatment period. Addition of exogenous TGFβ1 in the perfusate leads to the increased fluorescent intensity from both FITC (F-actin) and DAPI (α-SMA) channels. Verteporfin and TGFβ1 co-treatment attenuates the intensity of both FITC and DAPI channels.

HTCFs treated with TGFβ1 demonstrated increased proliferation and elevated presence of actin stress fibers compared to vehicle control (Figure 5). Interestingly, TGFβ1 treated HTCFs arrange themselves more uniformly with neighboring cells, suggesting organized ECM contraction. HTCFs cotreated with verteporfin demonstrated decreased area of fluorescence for F-actin and α-SMA resembling vehicle control. 

### 3.3. Afferent Line Pressure Changes

**Figure 6:**
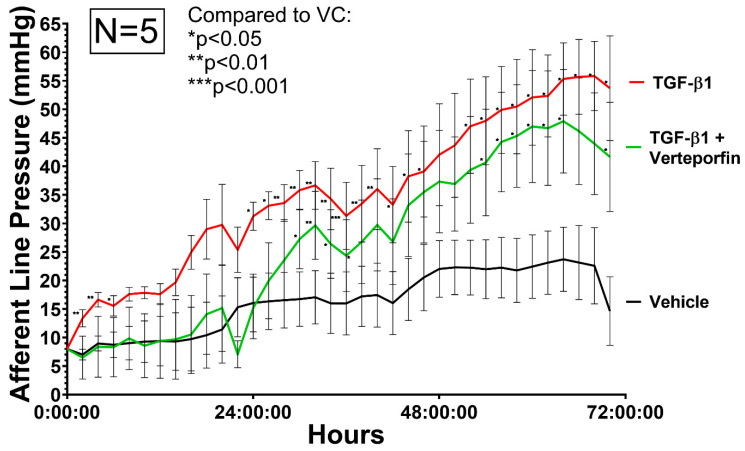
**Relative afferent line pressure is increased in HTCF subconjunctival tissue-mimetic treated with TGF-β1 (red).** Verteporfin (green) co-treatment decreased afferent line pressure. Pressure is measured in real-time with a blood pressure transducer representing model IOP. Relative pressure changes are normalized to average pressure values in the first hour of the experiment. Data shown are the means ± standard error (SEM) from N = 5 primary HTCF patient samples. (*) indicate significant differences between samples (* *p* ≤ 0.05; ** *p* ≤ 0.01; *** *p* ≤ 0.001) by 2 way ANOVA.

Initially, afferent line pressure was measured with an acellular collagen matrix, which demonstrated no pressure changes after initial system equilibration, and afferent line pressure remained constant for up to 72 h. Treatment of HTCF-containing collagen matrices with TGFβ1 demonstrated continual increases in afferent line pressure throughout the experimental duration (Figure 6). The difference in afferent line pressure between TGFβ1 only and VC was the greatest towards the end of the experiment, where the mean difference in afferent line pressure between ΔP_v_ and ΔP_t_ was 18.74 mmHg (SE = 5.726 mmHg). Significant pressure differences were achieved between VC and TGFβ1 after 26 h of perfusion. 

HTCFs co-treated with TGFβ1 and verteporfin demonstrated a significant delay in the generation of outflow resistance. The addition of verteporfin maintained afferent line pressure at levels similar to vehicle treated replicates up until 26 h after the start of the experiment. ΔP_vert_ demonstrated a mean afferent line pressure reduction of 8.51 mmHg (SE = 7.21 mmHg) compared to ΔP_t_. A two-way ANOVA revealed that there was a statistically significant difference between the effects of TGFβ1 and vehicle control (F(1, 8), *p* = 0.011). However, two-way ANOVA revealed that there was overall not a statistically significant difference between the effects of TGFβ1 and TGFβ1/verteporfin cotreatment (F(1, 8), *p* = 0.272) over the entire experimental duration.

### 3.4. Semi-Quantification of TGFβ1 Mediated Phenotypic Changes

Three-dimensional Z-stack confocal microscopy was performed and quantification of cytoskeletal fluorescence to the number of nuclei was compared between cells treated with TGFβ1, TGFβ1 and verteporfin, and vehicle control. Vehicle control cells expressed 49.4% of adjusted F-actin expression relative to TGFβ1 treated cells.

**Figure 7:**
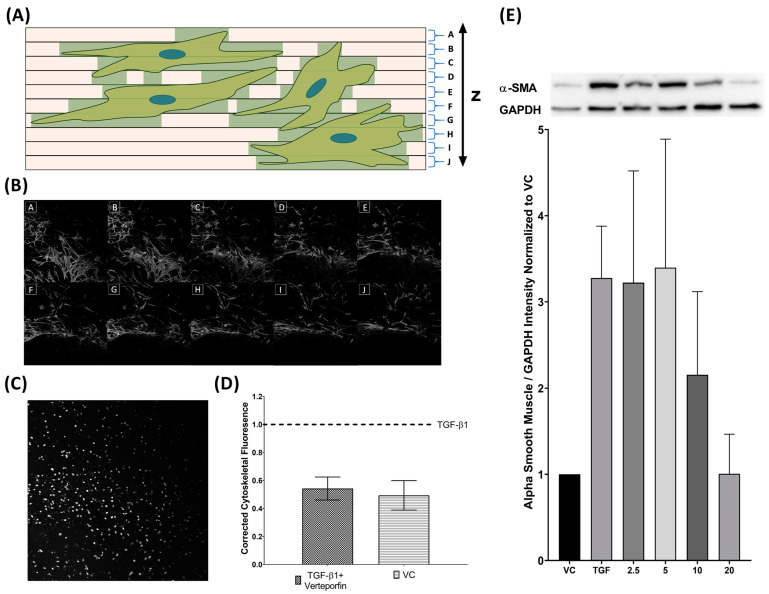
**TGFβ1 induced cytoskeletal proliferation is inhibited by verteporfin cotreatment.** Collagen slides are imaged as a Z-stack and divided in regular intervals from the bottom to the top of the slide (**A**). Cumulative cytoskeletal fluorescence (FITC) is summed to give total volume of fluorescence to account for the three dimensionalities of the cytoskeleton (**B**). Nuclei (DAPI) channels are projected onto a flat image and counted to quantify number of cells on each slide (**C**). Cytoskeletal fluorescence was normalized to the number of nuclei on the slide (**D**). Three technical replicates are performed for parallel runs of VC/TGFβ1 and TGFβ1/TGFβ1–verteporfin cotreatment. Verteporfin co-treatment also inhibits TGFβ1 mediated α-SMA (42 kDA) upregulation showing dose effect starting at 10 µM. α-SMA was normalized to GAPDH (36kDA) (**E**).

Cells treated with TGFβ1 and verteporfin demonstrated 54.3% of adjusted F-actin expression relative to TGFβ1 alone (Figure 7D). Concurrent Western blot revealed decreased levels of αSMA expression and demonstrated dose-response starting at 10 µM of verteporfin (Figure 7E).

## 4. Discussion

### 4.1. Development of a Three-Dimensional Flow Model

Three-dimensional tissue models more closely mimic the physiological milieu of living tissues, thereby circumventing many limitations inherent to 2D cell culture. Knowing that interstitial flow also affects tissue orientation and fibroblast transformation [26], the primary aim of the study was to develop a novel three-dimensional subconjunctival tissue mimetic and microfluidics platform in which potential novel antifibrotic compounds could be rapidly screened. One benefit of developing a model with interstitial flow is that as fibroblasts begin to transform, they begin to alter the properties of the hydrogel in which they were seeded in. Pressure changes can be appreciated with the pressure transducer upstream or by the height of media in the afferent line reservoir (Figure 3). In doing so, the functional implications of these drugs can be appreciated, as HTCF-mediated changes in overall outflow facility within the collagen matrix will directly affect afferent line pressure.

**Figure 8:**
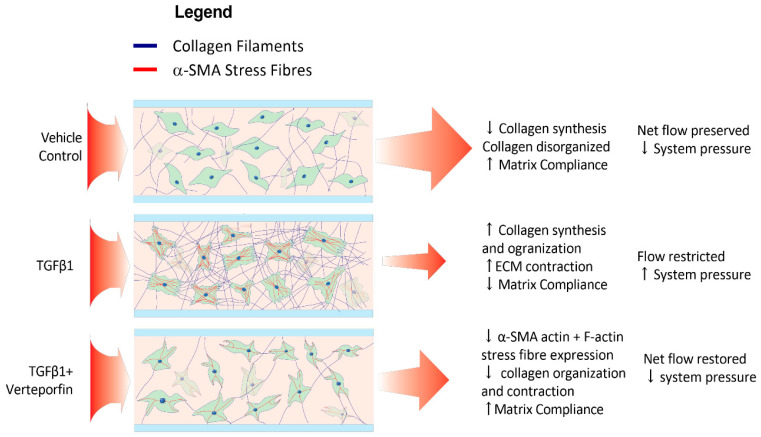
**Verteporfin attenuates TGF-β1-mediated changes within HTCFs in the hydrogel.** The net effect of TGF-β1 leads to myofibroblastic transdifferentiation indicated by increased cell bulk and α-SMA production. Myofibroblasts organize and contract ECM, lowering hydrogel permeability to perfusate and increasing system pressure. Verteporfin attenuates TGF-β1 mediated myofibroblastic transformation thereby lessening pressure increases.

The model was composed of loose ECM similar to what HTCFs would reside in vivo. This cellular hydrogel was then chemically locked to the inner surfaces of the flow chamber as the APTES and glutaraldehyde irreversibly crosslink the collagen onto the slide (Figure 2). Without this process, the collagen hydrogel would extrude through the system, and perfusate would flow around, not through, the HTCF–collagen suspension. Over the course of the experiment, fibroblasts within the hydrogel were integrated into the ECM. TGFβ1 naïve fibroblasts are capable of generating a moderate amount of outflow resistance within a collagen matrix as signified by afferent line pressure increases with the vehicle control group. This effect was dramatically increased with the addition of exogenous TGFβ1. The timeline over which afferent line pressure increased suggested that fibroblast transdifferentiation into myofibroblasts slowly changed the characteristics of the ECM, progressively increasing its resistance to outflow. As additional ECM proteins were synthesized and remodeled through myofibroblast activity, microscopic flow channels inside the hydrogel likely stenosed and overall tissue patency decreased, similar to the failing filtration bleb (Figure 8) [30,31].

### 4.2. Phenotypic Effects of TGF β1 Are Reflected within the Model

TGFβs have a major role in fibroproliferative disease [32,33]. Previous research has established that TGFβ2, another isotype, is found in high concentrations within the aqueous humor of patients with primary open-angle glaucoma and is implicated in instability in the blood–aqueous barrier [2]. Meanwhile, TGFβ1 is upregulated as a consequence of general wound healing as seen in surgical insult to tissue as well as neovascular glaucoma [34,35,36]. Both isotypes have been implicated in overexpression of genes responsible for ECM construction and stress fibre formation [37,38]. Canonically, TGFβ1 mediated SMAD2/3 activity contributes to the contraction of fibrillar collagen and inhibits metalloproteinases expression responsible for extracellular matrix (ECM) degradation [39]. Clinically, dysregulation of the aforementioned pathway has been implicated in fibrotic diseases in multiple organ systems [40,41].

In the presented model, exogenous TGFβ1 leads to increased myofibroblasts transdifferentiation consistent with previous work [27]. This was evident as TGFβ1 treatment alone was able to induce a-SMA upregulation (Figure 7E). Additionally, F-actin stress fibres, which allow tension generation and ECM remodeling, are elevated in HTCFs treated with exogenous TGFβ1 [42]. These biochemical findings correlate with the corresponding pressure data, as remodeling the hydrogel reduced the patency of the matrix to perfusate and reduce flow. As hydrodynamic resistance increased, the afferent pre-chamber pressure rose as perfusate began to accumulate. Overall, these results reaffirm that TGFβ1 alone can induce fibroblast transdifferentiation into its pathological myofibroblastic phenotype. More importantly, the clinical implications of these pathological changes were able to be captured by the microfluidics model through afferent line pressure increases. 

### 4.3. TGFβ1 Mediated SMAD2/3 Inhibition Leads to Attenuation of Fibroblast Transdifferentiation

Verteporfin is a benzoporphyrin derivative and first made its pharmacological debut as a photosensitizer in photodynamic therapy [43]. Recent work has shown that verteporfin is also a potent inhibitor of the YAP/TAZ signaling pathway [44,45]. Inhibition of YAP/TAZ augments inhibitory SMAD7 activity levels and prevents the SMAD2/3 complex from translocating into the nucleus to induce transcription of myofibroblast-related genes [46,47]. Furthermore, increased SMAD7 leads to matrix metalloproteases upregulation that is responsible for collagen degradation [48,49]. As discussed, the de novo deposition and contraction of the ECM are major contributors to surgical failure. It was demonstrated that YAP inhibition through verteporfin decreases contractility of trabecular meshwork cells [50], and previous reports have shown that verteporfin mitigates kidney myofibroblast transdifferentiation [51]. Likewise, this study revealed that verteporfin leads to decreased TGFβ1 mediated a-SMA expression (Figure 7E) and F-actin stress fibre formation (Figure 5) in HTCFs, suggesting that TGFβ1 mediated myofibroblast transformation is similarly inhibited in HTCFs. Furthermore, it was demonstrated that inhibiting myofibroblast transdifferentiation may have a tangible impact on outflow capacity as afferent line pressure increases were mitigated albeit only over the first 32 h of perfusion (Figure 6). Verteporfin photodegrades readily and has an estimated half-life of 5–6 h; in turn, its inhibitory effect could have been blunted as the experiment continued [52]. This is supported by the fact that the greatest mean difference in pressure was observed in the first 32 h of the experiment between TGFβ1 treatment and TGFβ1/verteporfin co-treatment.

This model does have several limitations relating to incomplete recreation of the physiologic milieu within the filtration bleb. Firstly, inter-patient and cell line variability leads to differing degrees of response to verteporfin leading to large standard deviation between runs. Despite this, the trend suggests that afferent line pressure is attenuated with verteporfin. Additionally, this fibroblast-only model is devoid of other immunomodulating cells. These cells help with cellular debris clearance and alter the activity of signaling molecules such as TGFβ1 in the subconjunctival tissues of the eye. Thus, future work incorporating THP-1 monocytes into this model as a co-culture with the fibroblasts within the collagen matrix is underway as macrophages are known to play a key role in wound healing. Finally, the length of each experiment was limited by how much volume was in the syringe pump as disconnecting to refill it with media would lead to system depressurization and potential contamination. Despite these limitations, proof of principle was established for this model. Future iterations of the model should incorporate a pneumatic pump system that allows for the continuous addition of new perfusate media without depressurization to provide uninterrupted perfusion of the system.

## 5. Conclusions

In this study, a three-dimensional HTCF collagen culture model was developed that permitted the perfusion of culture media and the ability to measure changes in afferent line pressure as an analogue for IOP. HTCFs in a collagen matrix were successfully used as an in vitro model to demonstrate the potential clinical implications of deranged TGFβ activity following a surgical-induced insult of the subconjunctival milieu. By incorporating a three-dimensional tissue mimetic, the model was able to reflect the behaviour of a filtration bleb while being able to monitor surrogate intraocular pressure when exposed to exogenous TGFβ1 as seen in post-surgical AH. Exogenous TGFβ1 increased afferent line pressure analogous to the clinical changes associated with impending surgical failure. The effects of TGFβ1 activity were able to be transiently attenuated following treatment with verteporfin. This model can serve as an in vitro platform for high-throughput screening of drug candidates that may potentially attenuate post-operative reductions to subconjunctival outflow capacity and pave the way for clinical translation and the benefit of future glaucoma surgery patients.

## Data Availability

All the information in the manuscript is supported by the mentioned references.
